# Role of Gut Microbiota in Neurological Disorders and Its Therapeutic Significance

**DOI:** 10.3390/jcm12041650

**Published:** 2023-02-19

**Authors:** Prabhakar Tiwari, Rekha Dwivedi, Manisha Bansal, Manjari Tripathi, Rima Dada

**Affiliations:** 1 Molecular Reproduction and Genetics Facility, Department of Anatomy, All India Institute of Medical Sciences (AIIMS), New Delhi 110029, India; 2 Department of Neurology, All India Institute of Medical Sciences (AIIMS), New Delhi 110029, India

**Keywords:** gut microbiota, neurological disorders, pathogenesis, therapy, gut–brain axis

## Abstract

In humans, the gut microbiota (GM) are known to play a significant role in the metabolism of nutrients and drugs, immunomodulation, and pathogen defense by inhabiting the gastrointestinal tract (GIT). The role of the GM in the gut–brain axis (GBA) has been documented for different regulatory mechanisms and associated pathways and it shows different behaviors with individualized bacteria. In addition, the GM are known as susceptibility factor for neurological disorders in the central nervous system (CNS), regulating disease progression and being amenable to intervention. Bidirectional transmission between the brain and the GM occurs in the GBA, implying that it performs a significant role in neurocrine, endocrine, and immune-mediated signaling pathways. The GM regulates multiple neurological disorders by supplementing them with prebiotics, probiotics, postbiotics, synbiotics, fecal transplantations, and/or antibiotics. A well-balanced diet is critically important for establishing healthy GM, which can alter the enteric nervous system (ENS) and regulate multiple neurological disorders. Here, we have discussed the function of the GM in the GBA from the gut to the brain and the brain to the gut, the pathways associated with neurology that interacts with the GM, and the various neurological disorders associated with the GM. Furthermore, we have highlighted the recent advances and future prospects of the GBA, which may require addressing research concerns about GM and associated neurological disorders.

## 1. Introduction

The gut microbiota (GM) are composed of microorganisms found in the gastrointestinal tract (GIT), such as archaea, bacteria, protists, and/or fungi [1]. It has been observed that the GM are150 times more genetically diverse than the human body, with 100 billion bacteria, ~1000 species, and ~three million genes [2]. Human growth, dietary requirements, physiological changes, and genetic variances are all impacted by individual GM, which have been found to be altered by age, gender, location, food, and genetic variations [3,4]. The GM are organized into four major phyla, including *Bacteroidetes, Firmicutes, Proteobacteria*, and *Actinobacteria*, and two minor phyla, including *Verrucomicrobia* and *Fusobacteri* [5]. These bacteria are known as commensal bacteria and communicate with each other in the host-gut epithelium in order to maintain gut homeostasis and to enhance host immunity [6]. The bidirectional transmission occurs in the gut–brain axis (GBA) in the form of a two-way communication mechanism between the gut and the neurological system of the host. This information can be transferred through the brain networks, hormones, and immune system, which facilitate the intestinal microbiota. The bidirectional transmission in the GBA regulates brain dysfunction mechanistically and also maintains a mutualistic association with the host and regulates the innate and adaptive immune systems [7,8]. The GM colonize the brain function during various cellular processes such as axonal processing, apoptosis, myelination, synaptogenesis, and cell differentiation and impairs cognition during neurogenesis when growth hormones, i.e., circulating insulin-like growth factor-1 (IGF-1), are present [9,10,11]. The long-term metabolic profiling of GM have revealed the regulatory function of brain development and its maturation impacts [12]. Around 200 million neurons are found in the central nervous system (CNS), which regulates the activity of the entire digestive tract. The CNS is composed of a network of nerve fibers and ganglia in the myenteric and submucosal plexuses [13]. The enteric nervous system (ENS) is found in the wall of the gastrointestinal tract (GIT) and is especially well-developed in the small and large intestines. It includes the submucosal (Meissner’s) and myenteric (Auerbach’s) plexus. These plexuses receive innervations from the vagus nerve and their activity is also regulated by pacemaker cells in the wall of the GIT and the interstitial cells of Cajal, which allow the plexus to function autonomously. Myenteric neurons, which are located between the inner circular and outer longitudinal muscles and can be either activating or inhibitory cholinergic neurons, aid in the peristaltic or myenteric reflex. The Meissner’s plexus is divided into two parts: inner and outer. The inner component, towards the muscularis mucosae, innervates the submucosal glands and the outer plexus innervates circular muscle and regulates peristalsis, blood flow, and digestive secretion. The myenteric plexus is responsible for gastrointestinal movement and is located deep between the longitudinal and circular layers of the digestive tract (peristalsis) [14,15]. Currently, the role of GM in the development of neurological disorders, neuro-inflammation, and neurobehavioral activities is well documented [9,16]. The GM play an important role in the pathogenesis and therapy of multiple neurological disorders such as Alzheimer’s disease (AD), multiple sclerosis (MS), Parkinson’s disease (PD), autism spectrum disorder (ASD), epilepsy, stroke, brain injury, amyotrophic lateral sclerosis (ALS), and Huntington’s disease (HD) [17,18]. Among the therapies, probiotics, postbiotics, prebiotics, symbiotic, antibiotics, and fecal transplantation have been investigated for GM-derived neurological disorders [19,20,21]. Henceforth, this review covers a detailed account of the role of GM in the GBA, which is associated in neurology pathways, and aspects of multiple neurological disorders together that have not been reported earlier. Furthermore, we have discussed the potential future implications of GM in neurological disorders, which may require the design of future research and address the pathological and therapeutic aspects.

## 2. The Role of GM in the GBA

Understanding the role of GM in the GBA for the neurodevelopment process is a growing trend in order to assess its impact on pathogenesis and therapeutic outcome. The gut communicates with the brain through two neuroanatomical pathways [22,23]. Firstly, in the spinal cord, the autonomic nervous system (ANS) and the vagus nerve (VN) are connected to the brain and the gut. Secondly, in GIT-ENS, bi-communication occurs via a bilateral connection between the gut and the brain. The ANS has three components: the sympathetic nervous system (SNS), the parasympathetic nervous system (PNS), and the enteric nervous system (ENS). The vagus is the tenth cranial nerve, arising from the medulla and emerging out of the skull through the jugular foramen. It has both general visceral efferent and afferent components and carries information to the brain from the GIT, heart, lung, and associated glands. It thus connects the brain to the GIT. It controls heart and respiratory rates, peristaltic activity, mood, and immune response. Recent studies have documented that stimulating the vagus nerves through yoga, which causes parasympathetic dominance and increases vagal tone, may be a potential treatment for refractory depression, post-traumatic stress disorder, and inflammatory bowel disease. Increasing the vagal tone also decreases cytokine production. The stimulation of the vagus nerve influences the monoaminergic brain system, which plays a crucial role in mood and anxiety disorders. The GM have anti-stress and anti-anxiety effects by influencing the activity of the vagus nerve and secreting neurotransmitters such as GABA, serotonin, and short-chain fatty acids [24,25,26,27,28].

### 2.1. Brain to Gut

It has been shown that the role of GM in invivo studies using animal models showed a lack of microbial colonization, which is linked to alterations in neurotransmitter synthesis [29,30]. All deficits were repaired when these animals colonized in a bacterial species-specific way while being impacted by higher doses of ACTH and cortisol. The germ-free (GF) animals reported minor anxiety and a higher stress response. Memory loss has also been linked to changes in the expression of brain-derived neurotrophic factor (BDNF) [31]. The use of some molecules has been demonstrated for the regulation of muscle repair, regeneration, differentiation, neural development, and cognitive functions [32]. The impact of probiotics or antibiotic therapy and the improved effect of enteric bacterial colonization on GBA were observed in the GM. GBA has been found to interact with bacteria to transmit information, which necessitates the existence of neurotransmitter receptors on bacteria. The role of GM is altered in the binding sites of enteric neurotransmitters, which are usually provided by the host [7,33]. The vagus nerve is occupied in the GBA and releases communication from the GIT to the brain for their functional activities [34].

### 2.2. Gut to Brain

It has been demonstrated that social stressors alter the profiles of the GM population and decrease the relative proportions of the major microbiota-associated phyla [35,36]. These stresses impact various factors such asmotility, acidity, bicarbonate, mucus secretion, intestinal fluid handling, and mucosal immune response, which are generated by the microbiome in the GBA. The GM aremodulated by the interference of the normal mucosal habitat, which is caused by GBA dysfunction [37,38]. Changes in intestinal permeability could potentially affect the structure and function of the microbiota [39]. Norepinephrine (released during surgery) has led to gut sepsis and altered the GM expression of *Pseudomonas aeruginosa* [40]. In addition, norepinephrine has been shown to stimulate the proliferation of many strains of enteric pathogens to increase their pathogenic features, such as *Campylobacter jejuni* and *Escherichia coli* [36,41].

## 3. Neurology Pathway in GBA

The neurotransmitter activity in the GIT, ENS, and vagus nerve is a component of the neurological route and stimulates the sensory nerves to release numerous hormones, including serotonin, melatonin, and histamine. It also releases GABA, acetylcholine, and catecholamines in the GIT [42]. The two pathways are discussed here, i.e., the endocrine pathway and the immune pathway.

### 3.1. Endocrine Pathway

In the endocrine pathway, enteric endocrine cells (EEC) release physiologically active peptides to affect the nutrient availability of the GM, which shows the association between nutrient sensing and peptide secretion by EECs and that this biologically active peptide alters the GBA [42,43]. EECs produce more than 20 peptides and/or hormones that act as signals for microbes, food borne toxins, nutrients, and non-nutrient toxins in the gut lumen and regulates nutrients absorption, intestinal immune response, and epithelial barrier defense [43,44]. EECs have an impact on food aversions andnutrient digestion and absorption, as well as defense mechanisms against toxins [45]. The secretory components of EECs are released into the bloodstream and use paracrine mechanisms to target the neuron [46,47]. Neurotrophin receptors promote the survival, growth, and function of neurons as well as pre- and post-synaptic proteins. The production of synaptic proteins raises the possibility of connections between EECs and nerves connecting the intestinal lumen via the ENS [48,49]. Galanin is an active peptide that affects sleep/wake, nociception, cell cycle control, appetite, mood, regulation of blood pressure, and parental and neurotrophic activities [50]. It also affects the production of the corticotrophin-releasing factor and the adrenocorticotropic hormone to activate the HPA axis centrally. Galanin increases glucocorticoid secretion in the adrenal cortex and releases norepinephrine in the adrenal medulla and cortisol from the adrenal cortex [51,52]. This implies its role in the hypothalamic–pituitary–adrenal axis (HPA)-mediated stress response.

### 3.2. Immune Pathway

Another important pathway is the immune pathway, which involves immunity via cytokine modulation in the intestine. Cytokine enters the bloodstream and is transmitted to the brain by the GBA [53]. Through the GBA, the immune system is known to be an important coordinator of microbiota and the brain [54]. Activation of the immune system in both the gut and the brain may lead to neuro-inflammation or neurological disorders that are activated by microbes with associated molecular patterns (MAMPs). These MAMPs are recognized by toll-like receptors (TLRs) to activate the various immune cells to generate pro-inflammatory cytokines such asIL1β, IL6, TNF-α, and IL17 that enter the brain through the blood–brain barrier and cause neurological disorders [55]. During dysbiosis in the GIT, the GM modulate the inflammatory metabolism, primarily by releasing various inflammatory cytokines, including IL-4, IL-10, and IFN-y, through their immune system [56,57]. Irritable bowel syndrome (IBS) is well defined by ENS dysregulation, which results in irregular microbial populations, activation of mucosal innate immune responses, increased gut epithelial permeability, and activation of gut sensory and epithelial permeability pathways [42,58]. The immune system’s influence on intestinal motility and secretion may result in visceral hypersensitivity and cellular entero-endocrine function abnormalities, as well as an impact on GIT and GBA functions [59]. Inflammasome activation leads to caspase-1 maturation and the release of pro-inflammatory cytokines IL1β and IL18 via specific MAMPs, resulting in a neurological disorder [60].

It has been shown that anti-TNF-α showsan association with a 78% reduction in PD [61]. The infection with *Citrobacter rodentium* in Pink1−/− mice promotes mitochondrial antigen presentation and auto reactive CD8 T cells and is found to be associated with an increase in motor impairment and PD brain pathology [62]. In addition, other results suggest that the association of microbes with the modulation of PD is mediated by an increased expression of TLR4 and CD3 in colonic mucosa and a reduced expression of tight junction markers [6]. It has been shown that in AD, amyloid-positive cells show an increased abundance of *Escherichia coli* and *Shigella,* which correlate with a systemic expression of IL1β, Nlrp3, and Cxcl2. The amyloid-positive cells have a reduced abundance of *Eubacterium rectale* and are found to be negatively associated with IL1β, Nlrp3, Cxcl2, and positively associated withIL10 [63]. The intestinal infection with *Helicobacter pylori* promotes pro-inflammatory innate and adaptive immune responses and shows a positive association with AD [64].

It has been observed that in a rodent model of experimental autoimmune encephalomyelitis (EAE), segmented filamentous bacteria (SFB) induces Th1 and Th17 responses during colonization in the intestine and spinal cord and leads to EAE symptoms in GF mice, while *Bacteroides* spp. and *Prevotella histolytica* colonization suppresses MS by promoting Treg function and suggests their association with Th17 cells [65,66]. It has been shown that the higher frequencies of Th17 cells in the small intestine are positively associated with *Streptococcus* and negatively associated with *Prevotella*, suggesting an increased and decreased level in MS patients, respectively [67]. An increased abundance of *Akkermansia* and *Acinetobacter* also induces inflammatory Th1 responses in MS patients. Additionally, the reduced abundance of *Parabacteroides* has beenobserved with induced anti-inflammatory T-reg responses in MS patients [68]. It has been observed that the FMT of healthy human microbiota show increased abundances of *Bifidobacteria* and *Prevotella* and improve the behavioral symptoms in ASD patients compared with their pre-transplant condition, but their immune association is unknown [69]. As a result, it has been demonstrated that regulating immune cell homeostasis is an alternative strategy for communicating microbes from the gut to the brain in the GBA.

## 4. Neurological Disorders and GBA

### 4.1. Parkinson’s Disease (PD)

Parkinson’s disease (PD) is caused by the deposition of α-synuclein (α-syn) on dopaminergic nerve cells in the substantia nigra, a part of the brain’s CNS. The α-syn is a defining feature of Parkinson’s disease and can be transported from the gut to the brain via the vagus nerve [70]. It has been shown that the truncal vagotomy of mice is protective against PD in the Danish and Swedish populations, whereas overall vagotomy or super-selectivity is not associated with PD, or a minor association is observed with PD, respectively [71,72]. The disease progression or pathogenesis of PD is altered with age, causes neurodegeneration, and modulates multiple cellular pathways [73]. PD is rare in people under the age of 50 and the chances of developing the disease are 5- to 10-fold higher at an older age. It affects mostly men and accounts for 5–35 new cases per 100,000 people each year [74]. Depositions of α-syn in the ENS have been linked to GIT abnormalities, which result in intestinal dysbiosis in PD patients [75,76]. The role of the GM has been shown to increase the possibility of physiological interactions between the host microbiome via cytokine networking PD [77]. Idiopathic constipation in PD is also linked to ENS neurodegeneration [78]. It has been shown that the stool samples showed a significant decrease in *Prevotellaceae species* when compared withthe relative counts of *Enterobacteriaceae* in the patients with PD [79]. In an invivo study using the PD model, short-chain fatty acids (SCFA) have been implicated as drivers of neuroinflammatory processes [80]. Using fecal transplantation therapy, the mice are significantly colonized with the microbiota of patients with PD and they acquire motor impairments and neuro-inflammation [81]. Antibiotic supplementation improves behavioral symptoms in PD patients. Intestinal microbial tyrosine decarboxylases have been shown to reduce the plasma levels of levodopa using the PD model in rats [79]. These findings indicate that changes in the GM play an important role in the pathogenesis and treatment of PD.

**Figure 1:**
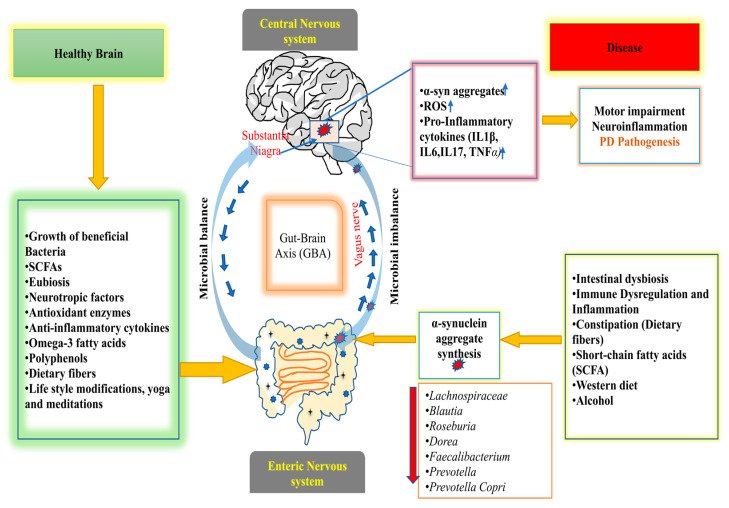
Depicts the role of GM in the GBA under microbial balance and imbalance conditions for the pathogenesis of PD. Microbial balance leads to a healthy brain (left side). It includes modifying factors such as beneficial bacteria growth, balanced SCFAs, eubiosis, increased neurotropic factors, the synthesis of anti-oxidant enzymes, anti-inflammatory cytokines, omega-3 fatty acids, polyphenols, and dietary fibers, which in turn keep the brain and biological system healthy, as well as lifestyle changes such as physical exercise, yoga, and meditation, in the intestine of the host. A microbial imbalance leads to the pathogenicity of PD (right side). It occurs due to intestinal dysbiosis, immune dysregulation and inflammation, constipation, decreased short-chain fatty acids (SCFA), the western diet, alcohol, etc. In PD, these factors promote the accumulation of α-synuclein, ROS, and pro-inflammatory cytokines (IL1β, IL6, IL17, and TNF-α), which are transported from the gut to the brain via the vagus nerve.

Figure 1 summarizes the role of GM in PD pathogenesis and their microbiota-associated modulatory effects.

### 4.2. Alzheimer’sDisease (AD)

Alzheimer’s disease (AD) causes memory loss and cognitive impairment due to the death of nerve cells; it is a type of dementia that typically affects people in their older age [82,83]. The accumulation of amyloid beta (Aβ) in the neurons and the de-phosphorylation of microtubules are associated with tau protein (t-protein) in the dendrites and axons of cortical neurons [84]. These biomarkers are hallmarks of AD [85,86]. The role of mitochondrial dysfunction is also an indication of AD, which affects the brain of AD patients and leads to aberrant mitophagy and affects mitochondrial quality control through mitochondrial malfunction and oxidative damage [87]. It has been shown that microbial infections such as *Chlamydia pneumonia,* fungal infections, and *spirochaetes* increase the level of pro-inflammatory cytokines in unstimulated and non-centrifuged blood and alter GM species profiling [88]. The presence of pro-inflammatory bacteria such as *Escherichia coli* and *Shigella* and anti-inflammatory bacteria such as *Escherichia coli* can cause dysregulation of the microbiota, which leads to systemic inflammation and aggravated neurodegeneration in patients with cognitive impairment and brain amyloidosis [89]. It has been shown that fumonisins (FBs), which are mycotoxins, produced by the fungus *Fusarium verticillioides* and administered in the GIT to rats, donot change the structure of the intestine but change the chemical code of the myenteric and submucosal neurons in terms of the neurochemical profile of enteric neurons. Fumonisins also impair neural development, including the B1 and B2 forms, and their role in a diet containing foods that inhibit myenteric neuron growth [90,91]. A combination of antibiotic treatments reduces the number of microglia and astrocytes around amyloid plaques. In persistent transgenic mice, the amount of insoluble amyloid plaques in the hippocampus is reduced [92]. The amyloid precursor protein (APP) in fecal samples shows significant variations in the GM assemblages compared withwildtype in the 16S rRNA sequence analysis using a transgenic mouse model [93]. In a study, toxic proteases such as gingipains are shown to be found in the brains of patients with AD and are associated with tau and ubiquitin pathology. Mice with oral *P. gingivalis* infection develop brain colonization and increase the production of amyloid –β (Aβ1-42), a substance seen in amyloid plaques. Inhibition of gingipain is important for the treatment of brain colonization of *P. gingivalis* and neurodegeneration. Additionally, it reduces the bacterial load during *P. gingivalis* brain infection and inhibits the synthesis of Aβ1-42, reduces neuroinflammation, and protectsneurons in the hippocampus [94]. A clinical research study was conducted in the United States involving a total of 108 elder people, of whom 51 had no dementia, 24 had AD, and 33 had other types of dementia. They performed metagenomic analysis using stool samples and an invitro assay for intestinal epithelial cells to see the expression of the P-glycoprotein protein with a follow-up of 5 months, which is an important modulator of intestinal homeostasis. They identified a microbial taxon in which *Bacteroides* spp., *Alistipes* spp., *Odoribacter* spp., and *Barnesiella* spp. are more common and *Lachnoclostridium* spp.isless common. The *Butyrivibrio* genus and other bacteria that have the ability to produce butyrate are less common and proportionately less prevalent in the AD microbiome. In addition, this study suggests that AD microbiota may negatively impact intestinal epithelial homeostasis by dysregulating the P-glycoprotein pathway [95]. The GM inflammasome proteins are produced at higher levels and serve as an important precursor for the activation of downstream cytotoxic and inflammatory mediators. The gastrointestinal inflammasome NLRP3 protein may increase neuro-inflammation, implying that GM modification could be a vital treatment for AD-related genetic predisposition in neurological disorders [96]. In one study, fecal short-chain fatty acids (SCFA) and microbial composition at various ages reveal a significant decrease in *Butyricoccus* and *Ruminococcus* and an increase in *Proteobacteria* and *Verrucomicrobia* in AD mouse models compared withwild type. These changes in microbial composition and diversity, as well as decreases in SCFA levels, suggest a disruption of at least 30 metabolic pathways [97]. It has been shown that the intake of probiotic bacteria and dietary modifications such as ketogenic diets are useful to prevent the progression of AD [98]. These results implicate the role of GM in AD and could show how GM influence the pathophysiology and treatment of the disease.

**Figure 2:**
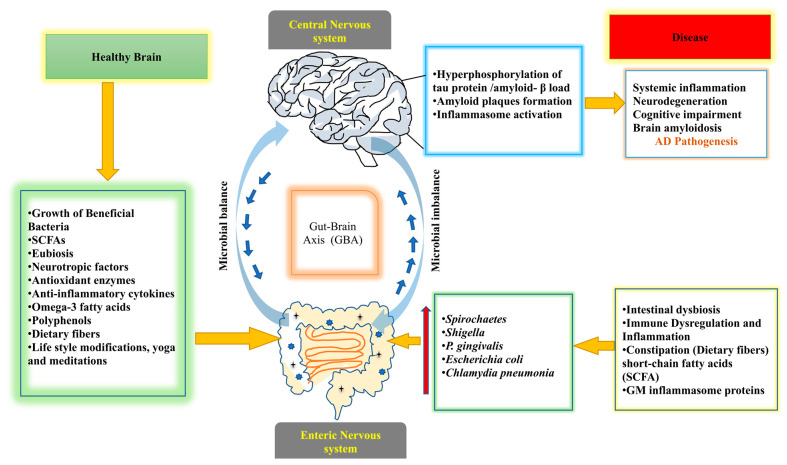
Demonstrates the role of GM in AD pathogenesis via GBA in both microbial balance and imbalance conditions. A healthy brain (left side) occurs due to a microbial balance condition in the intestine. It is affected by various factors, including the growth of beneficial bacteria, balanced SCFAs, eubiosis, increased neurotropic factors, anti-oxidant enzyme synthesis, anti-inflammatory cytokines, omega-3 fatty acids, polyphenols, dietary fibers, and lifestyle changes such as yoga and meditation;all of these factors contribute to brain health. A pathogen that causes Alzheimer’s disease when there is a microbial imbalance (right side). It may cause intestinal dysbiosis, immune dysregulation and inflammation, constipation (dietary fibers), decreased short-chain fatty acids (SCFA), etc. GM inflammasome proteins cause tau protein hyperphosphorylation and amyloid-load plaque formation in the brain, as well as inflammasome activation, resulting in systemic inflammation, neurodegeneration, cognitive impairment, and brain amyloidosis, thus leading to AD pathogenesis.

Figure 2 summarizes the role of GM in AD pathogenesis and the modulatory effects on the GBA.

### 4.3. Multiple Sclerosis (MS)

Multiple sclerosis (MS) features damaged axons, demyelination, and immune-mediated dysfunction; it affects ~2.3 million populations worldwide, with a higher female prevalence [99]. The formation of demyelinated plaques is found in either the grey or white matter of the spinal cord and brain and induces a neuro-inflammatory response that results in the demyelination of specialized cells such as neurodegeneration and oligodendrocytes [100]. Experimental autoimmune encephalomyelitis (EAE) in a mouse model indicates that CD4+ T cells play an important role in the pathogenesis of MS [101]. The GM has been associated with the pathophysiology of MS and its modulation has been demonstrated in an invivo study using EAE and validated animal models. Additionally, it has been shown that interleukin-10-producing CD4+ T cells play an important role in immune modulation activities [26,68]. According to one study, gram-positive segmented filamentous bacteria in the GI tract activate Th17 cells and have a significant impact on EAE severity [102]. The microbiota have been linked to myelin synthesis regulation in MS in GF mice and pre-clinical antibiotic treatments in the mouse pre-frontal cortex. A study on the breakdown of the blood–brain barrier (BBB) in GF animals has demonstrated that the GM are involved in maintaining the BBB’s integrity [103]. Diet induction has been linked to changes in GM composition in EAE [104]. Using the multi-species probiotic (*Lactobacillus species, Bifidobacterium species*, and *Streptococcus species*) for two months and twice a day has been shown to counteract microbial changes and have anti-inflammatory features [105]. These findings from animal and human clinical studies suggest that GM have a significant impact on MS physiopathology [106]. However, the current focus is on how to successfully modify the GM as an intervention to prevent relapse and symptoms of MS to a greater extent and to cure the disease.

### 4.4. Autism Spectrum Disorder (ASD)

Autism spectrum disorder (ASD) is a neuro-developmental disorder characterized by difficulties in social interaction and communication, as well as repetitive behavior patterns [107]. According to the CDC’s Autism and Developmental Disabilities Monitoring (ADDM) network, one out of every 44 children is affected by ASD [108]. ASD is caused by a variety of factors, including malnutrition, infections, and developmental disorders in infancy, as well as maternal auto-antibodies against -7proteins in the developing brain [109,110]. Recent research has shown that GM and brain interactions may impact autism, a neuropsychiatric disorder. In addition, ~40% of people with ASD have more gastrointestinal problems [107,111]. In a study, it has been shown that GM affect mood and behavioral changes from childhood to adulthood [112]. The microbiome colonizes the gut immediately after birth and connects to the brain as the child grows. During the development process, any inflammation or impediment results in impaired cognition, changes in mood and memory, and leads to atypical behavior [113]. The epidemiologically established risk factors such as maternal exposure to the anti-convulsant valproate, maternal inflammation during pregnancy, and maternal obesity altered the GM composition in the ASD animal model [114,115]. ASD has been associated with GM species that are vulnerable to vancomycin and produce a pro-inflammatory condition [116]. Probiotics (live microbial cultures that are beneficial to the host) and/or prebiotics (non-digestible carbohydrates such as fibers that are beneficial to the host and/or microbiota) have been shown to modulate animal social behavior [117]. These findings are intriguing because they can be applied to humans and could lead to novel microbiota-based therapies for ASD treatment.

### 4.5. Stroke and Brain Injury

Currently, stroke and brain injuries are major causes of morbidity and mortality worldwide. They may occur due to modifications in various diseases such as cerebrovascular disease, atherosclerosis, dyslipidemia, diabetes, arterial hypertension, etc. Acquired brain injury (ABI) comprises two forms, i.e., traumatic brain injury (TBI) or non-traumatic brain injury (non-TBI), and their patients need primarily advanced pre-hospital treatment, comprehensive clinical care, and long-term recovery; therefore, certain neuroprotective or neurorestorative strategies or therapies are required to protect the brain from injury [118]. In addition, the commensal bacteria of the GM microflora may be involved in the development of stroke and/or brain injuries [119,120]. Cerebral ischemia is linked to altered GM composition as well as GIT effects on motility and barrier permeability. It has been shown that the transplantation of fecal microbiota into GF mice or from stroke patients into antibiotic-treated mice worsens the ischemia-induced cerebral lesion volume and is associated with functional deficits in a pre-clinical study [121,122]. Antibiotic-induced microbiota dysregulation reducesIL-17 cytokine trafficking in T cells as well as chemokine production. GM affect intestinal T-cell trafficking to the brain and alter the degree of neuroinflammation, which can lead to a stroke or brain injury. Using a broad spectrum of antibiotics for the middle cerebral artery blockage worsens the survival rate in mice. In addition, the induced GI microflora reduce IL-17 migration into YδT cells and the production of associated chemokines and regulate intestinal T-cell infiltration into the brain, which modulates neuro-inflammation after a stroke [123]. It has been shown that a microbiota-derived metabolite (trimethylamine n-oxide (TMAO), produced from dietary choline) is associated with the risk of cerebrovascular and cardiovascular disorders and suggests their role in disease pathogenicity [124,125,126]. The phosphatidylcholine metabolites (choline and TMAO) are shown to be involved in atherosclerosis due to the presence of bacteria in the gut. The healing of atherosclerotic lesions is significantly aided by a healthy microbiome [127]. Patients suffering from transient ischemic attack or stroke have been found to have opportunistic pathogens such as *Desulfovibrio, Enterobacter, Megasphaera*, and *Osicillibacter,* as well as fewer beneficial or commensal pathogens such as *Bacteroides, Fecalibacterium, and Prevotellz* [128]. The abundance of *Peptococcaceae* and *Prevotellaceae* is linked to stroke severity. The precise role and mechanism of GM in the onset and progression of stroke and brain injury remain unknown. Although animal models have yielded fascinating results, more clinical research is needed to fully elucidate the potential of such microbial therapeutic modalities.

### 4.6. Epilepsy

Epilepsy is a chronic neurological disorder that affects ~65 million people worldwide. Currently, despite being on anti-epileptic drug (AED) medication, only 70% of individuals with epilepsy achieve complete seizure control. As a result, around one third of epilepsy patients have refractory seizures that interfere with daily activities [129]. It has a significant socio-economic impact due to a higher morbidity and mortality rate, indicating an urgency to develop more effective, curative, and potential treatments [130]. It has been discovered that epilepsy is linked to intestinal bacterial species, implying that GM can help treat epilepsy [131]. The microbial communities are found in healthy gut microflora and have demonstrated their impacts on both pro- and anti-inflammatory conditions, which suggest that the immune system has been linked to a balanced gut microflora. It has been shown that chronic inflammation leads to the onset and progression of epilepsy and, further, GM can modulate immune and inflammatory responses [132,133]. Manipulation of GM diversity may be a viable treatment method, as demonstrated by differences in GM profiling using multiple therapeutic approaches for uncontrolled epilepsy compared with a healthy population [134]. It has been shown that *Firmicute* bacteria are capable of controlling neurotransmitter levels and larger numbers of *Lactobacillus* and *Bifidobacterium* are associated with a lower number of seizures per year [135]. The α-diversity in GM is found to be significantly higher in drug-resistant patients than in drug-responsive patients who are similar to healthy controls. Higher levels of α-diversity are associated with a large number of rare intestinal bacterial species, with significant differences observed at the genus level, indicating that bacteria may play an important role in epilepsy therapy. The intestinal GM act as an anti-epileptic medication to modify zonisamide metabolism. The ketogenic diet (KD) is found to reduce seizure frequency in epilepsy patients and change the composition and structure of GM in dietary therapy [136]. A KD mediates the anti-seizure effects in the temporal lobe of epilepsy in the GF mouse model and the increased seizure threshold has been observed with transplantation of KD microbiota species such as *Akkermansia muciniphila, Parabacteroides distasonis*, and *Parabacteroides merdae* [137]. In comparison to other diseases, there is only limited evidence for the role of GM in epilepsy. Therefore, researchers should focus on the microbiota’s ability to affect the physiology and behavior of epilepsy disorders.

### 4.7. Amyotrophic Lateral Sclerosis (ALS)

Amyotrophic lateral sclerosis (ALS) is a severe late-onset neurodegenerative disorder that affects motor neurons and affects nearly one person in every 1000. The vast majority of ALS cases are sporadic, with familial ALS accounting for only 5–10% of all cases. The sporadic and familial ALS (FALS) cases have shown cortical and spinal motor neuron degeneration, which may occur due to mutations in superoxide dismutase-1 [138]. The GM are an important part of our bodies’ landscape, performing inter-individual changes and impacting ALS [139]. The anti-microbial peptide (defensin -5) has been shown to be lower in the gut. These changes in G93A mice are due to an altered microbiome profile, i.e., lower amounts of *Butyrivibriofibrisolvens, Escherichia coli*, and *Fermicus* compared with wild-type mice. This result suggests that the intestinal epithelium and microbiome could play an important role in ALS development [140]. The GM are altered with a lower relative abundance of butyrate-producing bacteria compared with healthy mice in a mouse model of ALS, which is attributed to changes in the gut permeability [141]. ALS patients have different gut microbial communities compared with healthy controls, which suggest that manipulating the GM, e.g., addressing *Prevotella* spp. deficiency or changing butyrate metabolism, could be useful in the treatment of ALS [142]. In a clinical study, the microbial structure with *Bacteroidetes* (at the phylum level) and numerous bacteria (at the genus level) is up-regulated. However, *Firmicutes* (at the phylum level) and *Megamonas* (at the genus level) are down-regulated in ALS patients using 16S rDNA sequencing compared with healthy controls. In addition, ALS patients have lower gene function in metabolic pathways. These findings indicate that GM and metabolic products are possible therapeutic targets that should be investigated in future studies [143]. In a prospective longitudinal study, the probiotic supplementation impacts the GM, which is associated with the progression of ALS. The imbalance phenomenon has been observed between protective microbial groups (*Bacteroidetes*) and neurotoxic or pro-inflammatory activities (*Cyanobacteria*). It has been shown that the GM composition is changed with six months of probiotic treatment, although no effect is found during the course of the disease, and the diversity of the intestinal microbiota of patients is not restored like that of healthy controls [144]. These studies reveal the impact of GM in ALS, but more clinical research evidence is still needed to be evaluated in humans to establish the exact role of microbiota in the pathogenesis of AML.

### 4.8. Huntington’s Diseases (HD)

HD is a neurodegenerative autosomal dominant disorder and is marked by the clinical symptoms of progressive motor, cognitive, and psychiatric disturbances and sudden weight loss [145]. The trinucleotide (cytosine–adenine–guanine, CAG) repeat in the Huntington (HTT) gene is expressed in the brain and an expanded CAG repeat length is the main cause of HD. In addition, other genetic factors such asoxidative stress, DNA damage, mitochondrial dysfunction, neuroglia dysfunction, and protein aggregation lead to HD [146,147]. Recent studies have suggested that HD may be associated with gut dysbiosis, which is caused by GIT dysfunction in an HD mouse model. In an invivo study of the HD mouse model, a significant difference in GM composition at 12 weeks is observed and found to be due to an increase in *Bacteroidetes* and a decrease in *Firmicutes* communities [148]. It was discovered that patients with HD have a significant difference in GM composition in terms of increased β-diversity and decreased α-diversity (richness and evenness) when compared with healthy controls [145]. These research findings show a strong link between GM and cognitive ability and clinical outcomes in HD. However, more research is needed to fully understand the role of the GM and their metabolites in disease progression and the severity of behavioral disorders in HD for curative purposes.

## 5. Future Prospective

**Figure 3:**
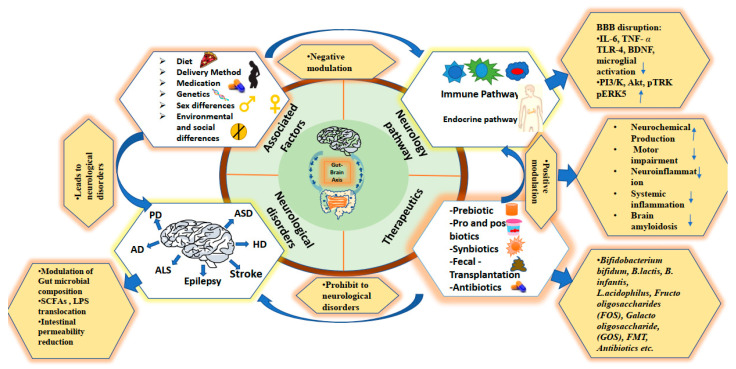
Shows an overview of the neurological disorders associated with GM in GBA. This figure illustrates the associated factors that cause the neurological disorder and the pathways involved, which are modulated by factors (negatively modulated) or therapeutics (positively modulated). The dysregulation of these pathways causes several neurological disorders such as Parkinson’s disease (PD), Alzheimer’s disease (AD), multiple sclerosis (MS), amyotrophic lateral sclerosis (ALS), autism spectrum disorder (ASD), stroke and brain injury, epilepsy, and Huntington’s disease (HD). In addition, we have provided information on microbial treatments that are being utilized to treat neurological disorders, including prebiotics, probiotics, postbiotics, synbiotics, antibiotics, and fecal transplantations. The disease-associated modulation of biomarker levels, as well as the micro-organisms involved in therapeutics and their beneficial effects to prevent the disease, are also represented.

Major populations are suffering from neurological disorders, which are expected to rise by 13% by 2030. Hence, there is an urgency to develop more reliable biomarkers and feasible therapeutic options in view of the diseases’ pathogenicity. In this context, the GBA is a fascinating research area for understanding the role of the GM and has gained research interest in recent decades. The microbiome, which includes archaea, bacteria, protists, and fungi, resides in and on our bodies and plays a significant role in the GIT’s microflora; its unbalancing is linked to the cause of specific aberrant physiological situations, highlighting the importance of the GBA microbiota for an individual’s health. A summary of the function of GM in neurological disorders and their modulatory effects on the GBA are represented in Figure 3. Various factors may have contributed to the modification of GM in the GBA and have led to a variety of neurological disorders that modulate neurologically associated pathways. The unbalancing of GM and their dysfunctional activities can be regulated by the administration of different treatments, such as probiotics, prebiotics, synbiotics, postbiotics, antibiotics, and fecal transplantation, which alter the composition or function of the GM in the brain. Multiple studies show that the GM are critical for brain development and function. In a number of pre-clinical and clinical research studies, it has been shown that the GIT microbiome in the GBA has been reviewed for the association of multiple neurological disorders such as AD, MS, PD, ASD, epilepsy, stroke and brain injury, AML, HD, etc. [9,12,23,149]. However, deeper research is needed for the understanding of the mechanism of the action and function of GM in disease pathogenesis and its further applicability for therapeutic or prognostic purposes. The GM’s composition and their metabolites disrupt the immunological and endocrine systems of the host, impacting brain function and blood flow. Currently, a number of studies are based on correlation rather than causation. To prove causation, more prospective studies are needed. Another concern is that a number of studies have been published that are performed with animal models and limit the findings to human studies. Additionally, several confounding factors connected with human fecal research, including food, demographic, clinical, and socio-economic characteristics, as well as sample collection, laboratory methods, and genetic sequencing techniques, are likely to contribute to the multiplicity of research findings. Researchers are needed to conduct such research, which can help in determining more thorough causes and the impacts of underlying pathways by using interventional approaches such as the use of probiotics, prebiotics, fecal transplantation therapy, etc. There are various concerns and difficulties associated with routinely using microbial therapies, such as FMT, probiotics, and prebiotic supplements, in the prevention and treatment of neurological disorders [150,151]. Additionally, it is critical to take into account the appropriate dosages of probiotics and other microbial therapies, as the ideal dosages and lengths of treatment have not yet been fully elucidated. Pre-clinical and clinical trials for probiotics and other microbial therapies differ significantly in the timing, formulation, and dosage of treatments for neurologic disorders. The disease-indicative target population for microbial therapeutic intervention must be identified for the ideal stage of the disease and the patient’s age [152]. To further develop therapeutic strategies, it is necessary to establish the effects of food components and metabolites generated by microbes on host physiology and health [153,154]. Our previous studies on yoga and meditation and yoga-based lifestyle interventions have shown that they can modulate markers of stress in the serum (cortisol, interleukin-6, brain-derived neurotrophic factor (BDNF), DHEA, and reactive oxygen species) at 6–8 weeks post-intervention compared with the baseline on the assessment of glaucoma and retinoblastoma patients. These interventions improve oxidative stress and quality of life [155,156,157]. In addition, some studies have documented the beneficial effect of yoga on the brain [158,159]. However, the impact on GM and the composition of their beneficial species in GBA still need to be elucidated in future studies. Because many patients are given multiple medications, more research is needed to clarify any potential GM–drug interactions. The GM are new lines that separate human health from a variety of disorders and future neurotherapeutic research will provide critical information on this topic. In spite of recent developments in our understanding of the GBA, further research is required to determine whether or not this knowledge can be helpful in a clinical environment. Future studies must clarify the underlying links between the GM and various neurological diseases and determine whether or not treating the microbiota is a safe and effective course of treatment.

## 6. Conclusions

Currently, the role of gut microbes in neurological disorders with GBA is evolving in research trends to obtain attention for its future scope in the scientific community. Despite the fact that numerous research studies are being conducted to determine the precise role and mechanism of GM with GBA for neurological disorders, its therapeutic implications also remain to be evaluated and established for human health preservation. The paradigmatic alterations in the GM or disruptions in the microbiome associated with the GBA directly or indirectly impact brain function. The microbiotas of each individual are unique and vary widely from person to person. The microbiome immediately begins to colonize the gut after birth and it connects with the brain during development. An imbalance in the beneficial interactions between the GM and normal physiology results in dysbiosis. The microbiotas in the gut are impacted by food, inactivity, and medications. Early detection of neurological disorders can be aided by research into changes in the GM and revealing them for further prognostic or therapeutic interventions. The GM and their metabolites are modulated by various factors and their imbalances by the host’s immune and endocrine systems and lead to the development of neurological disorders. Through bacterial metabolites, neurotransmitters, IECs, and the immune system, the GM regulates neurophysiological function and cognition. Currently, the association between the GBA and immune-mediated neurological disorders is gaining attention, and bi-directional communication, including gut-to-brain and brain-to-gut, needs to be addressed concerning the complications of the BBB.

Furthermore, there are many confounding factors associated with human microbiota that are altered by disease specificity and may be changed by the associated heterogeneity of findings. These factors include diet, lifestyle modifications, demographic, clinical, and socio-economic factors, which are also dependent on sample collection and laboratory procedures and the types of high-tech genetic sequencing approaches that should be selected in a very precise manner. Currently, the role of the GBA in neurological disorders is needed to identify neurodegeneration biomarkers and to develop novel treatment modalities such as probiotics, prebiotics, postbiotics, synbiotics, antibiotics, and fecal transplantation. Despite recent advances in evidence in the GBA, more data are needed to address mechanisms and to gather the knowledge of disease-associated pathogenesis that can be utilized in the clinical setting. Henceforth, future research needs to establish more clear causal relationships between the microbiota and different neurological disorders to establish the use of the microbiota as a safe and beneficial therapeutic option. Apart from correlative studies, novel advanced technological approaches are needed to identify those that can validate mechanisms of action and that may be able to develop treatment modalities for neurological disorders. Therefore, we emphasize that a deeper understanding of the microbiota and the GBA may aid in the progression of disease pathogenesis and treatments to improve brain function for various-noted neurological disorders. We may expect that GM-based therapy will be able to provide more promising prognostic or therapeutic approaches to treating neurological disorders in the future.

## Data Availability

Not applicable.
